# Optimal Immobilization of ****β****-Galactosidase onto ****κ****-Carrageenan Gel Beads Using Response Surface Methodology and Its Applications

**DOI:** 10.1155/2014/571682

**Published:** 2014-02-02

**Authors:** Magdy M. Elnashar, Ghada E. Awad, Mohamed E. Hassan, Mohamed S. Mohy Eldin, Bakry M. Haroun, Ahmed I. El-Diwany

**Affiliations:** ^1^Polymer Department, National Research Centre, El-Behouth Street, Dokki, Cairo 12311, Egypt; ^2^Centre of Scientific Excellence, Group of Biopolymers and Nanobiotechnology, National Research Centre, El-Behouth Street, Dokki, Cairo 12311, Egypt; ^3^Chemistry Natural & Microbial Products, National Research Centre, El-Behouth Street, Dokki, Cairo 12311, Egypt; ^4^Polymer Department, Advanced Technology and New Materials Research Institute, City of Scientific Research and Technological Applications, Borg Al-Arab, Alexandria 21934, Egypt; ^5^Faculty of Science, Al-Azhar University, Cairo 11651, Egypt

## Abstract

**β**-Galactosidase (**β**-gal) was immobilized by covalent binding on novel **κ**-carrageenan gel beads activated by two-step method; the gel beads were soaked in polyethyleneimine followed by glutaraldehyde. 2^2^ full-factorial central composite experiment designs were employed to optimize the conditions for the maximum enzyme loading efficiency. 11.443 U of enzyme/g gel beads was achieved by soaking 40 units of enzyme with the gel beads for eight hours. Immobilization process increased the pH from 4.5 to 5.5 and operational temperature from 50 to 55°C compared to the free enzyme. The apparent *K*_*m*_ after immobilization was 61.6 mM compared to 22.9 mM for free enzyme. Maximum velocity *V*_max_ was 131.2 **μ**mol*·*min^−1^ while it was 177.1 **μ**mol*·*min^−1^ for free enzyme. The full conversion experiment showed that the immobilized enzyme form is active as that of the free enzyme as both of them reached their maximum 100% relative hydrolysis at 4 h. The reusability test proved the durability of the **κ**-carrageenan beads loaded with **β**-galactosidase for 20 cycles with retention of 60% of the immobilized enzyme activity to be more convenient for industrial uses.

## 1. Introduction

Lactose is the main carbohydrate contained in milk at a concentration between 5% and 10% (w/v) depending on the source of milk [1]. Lactose could be also found in whey permeate at higher concentrations. The consumption of foods with a high content of lactose is causing a medical problem for almost 70% of the world population, especially in the developing countries, as the naturally present enzyme in the human intestine loses its activity during lifetime [2]. Unfortunately, there is no cure to lactose intolerance. This fact, together with the relatively low solubility and sweetness of lactose, has led to an increasing interest in the development of industrial processes to hydrolyze the lactose contained in dairy products (milk and whey) with both the free and immobilized conditions [3]. The studies have shown that glucose and galactose, two monosaccharides hydrolyzed from lactose, are four times sweeter than lactose, more soluble, and more digestible [4] and can be consumed by “lactose intolerant” people [1, 5]. Hydrolysis of lactose present in whey permeate will produce lactose-free syrup, solving an aquatic pollution problem as whey is usually thrown in water.

Immobilized enzyme is more favorable than free enzyme since it offers the possibility of continuous flow processing, so that easy regeneration of the immobilized enzyme and low cost operation can be achieved in industrial processing.

Many techniques have been used previously for enzyme immobilization, including entrapment [6], cross-linking [7, 8], adsorption [9], or a combination of these methods [10]. *β*-Galactosidase has been immobilized onto a wide variety of solid supports such as Sephadex, alginate, *κ*-carrageenan, chitosan, porous glass, agarose, polyvinyl alcohol polymers, diethylaminoethyl cellulose, Eupergit C (epoxy-activated acrylic beads), nylon, polyurethane foams, or zeolite [11]. Carrageenan has been used for the immobilization of enzymes and cells using entrapment techniques. It is inexpensive but suffers from weak mechanical and thermal stability [12]. Some work has been performed to improve its mechanical and thermal stability and it was found that the gels mechanical strength increased with the increased addition of 3,6-anhydro-D-galactose 2-sulphate in the polymer or after addition of gums. K^+^, Al^3+^ was also found to improve the gels characteristics. In the field of immobilization of enzymes, *κ*-carrageenan is one of the main supports used for cell and enzyme immobilization via entrapment; for example, *κ*-carrageenan was used to immobilize *α*-chymotrypsin using an encapsulation method. However, one of the main disadvantages of these biopolymers is that they are usually used for immobilization of enzymes using noncovalent bonds (mainly entrapment/encapsulation) due to the lack of functionalities. Unfortunately, the entrapment of enzymes in hydrogels is often characterized by some diffusion of the biocatalyst from the support, particularly for enzymes with molecular weight less than 300 kDa. Recently, a few authors were successful at covalently immobilizing enzymes using hydrogels [13–15].

To overcome the problem of the gels' low thermal stability, gels were treated with polyamines to form a polyelectrolyte complex. According to [16] the thermal stability of *κ*-carrageenan gels could be improved by adding amine compounds and, especially, polyamine compounds. For this reason, natural polyamines such as chitosan and synthetic ones such as polyethylenimines were used to improve the carrageenan gels' thermal stabilities [17].

## 2. Materials 

*κ*-Carrageenan (Mw, 154,000; sulfate ester 25%) and glutaralde-6-hyde solution (GA) (25%) were purchased from FLUKA (Switzerland). Glutaraldehyde solution (GA) (25%) was purchased from FLUKA (Switzerland). Polyethyleneimine (PEI) was obtained from Sigma-Aldrich (Germany). Lactose is from Arabian medical & Scientific Lab, Dubai, UAE, and *β*-galactosidase (EC 3.2.1.23) from *Aspergillus oryzae*, 11.8 U/mg, was purchased from Sigma-Aldrich. Other chemicals were of Analar or equivalent quality.

### 2.1. Experimental Techniques

As a general rule, experiments were carried out in triplicate and data are means ± SD (*n* = 3) unless stated otherwise.

## 3. Methods

### 3.1. Determination of *β*-Galactosidase Activity

*β*-Galactosidase activity was determined by the rate of glucose formation in the reaction medium. Known amount of immobilized or free enzyme was incubated into 10 mL of 200 mM lactose solution in citrate-phosphate buffer (100 mM, pH 4.5) for 3 h at 37°C. At the end of the time 50 *μ*L of reaction mixture was added to 950 *μ*L buffer and boiled for 10 min to inactivate the enzyme and analyzed for glucose content using the glucose test. One enzyme unit (IU) was defined as the amount of enzyme that catalyzes the formation of 1 *μ*mol of glucose per minute under the specified conditions [17].

### 3.2. Preparation of Carrageenan Beads

**Figure 1:**
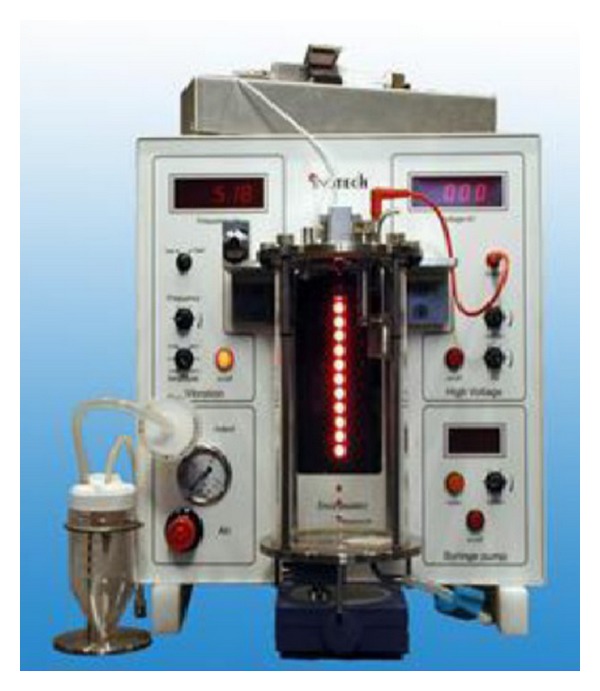
Encapsulator for making uniform gel beads.

*κ*-Carrageenan gel was prepared as previously reported by Elnashar and Yassin [17] by dissolving 2.5% (w/v) carrageenan in distilled water at 70°C and 0.002% (w/v) NaN_3_ was added as antibacterial using an overhead mechanical stirrer until complete dissolution had occurred. Then carrageenan gel was transferred to an encapsulator (Figure 1) to make gel beads. The carrageenan gel beads were hardened using 0.3 M KCl for 3 h.

### 3.3. Activation of Gel Beads

**Scheme 1:**
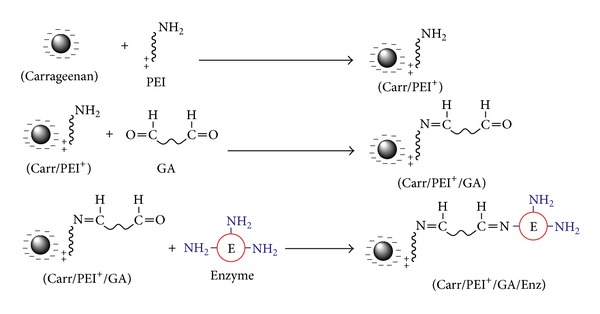
Mechanism of activation of carrageenan beads and enzyme immobilization.

For activation of carrageenan beads, they were soaked in polyethyleneimine (PEI) at desired concentration and left to react with it at room temperature. The unreacted PEI was then removed from the beads by successive washing with distilled water. The aminated beads (PEI-carr.) were then reacted with glutaraldehyde (GA) solution of specific concentration for selected time and temperature, and then the beads were washed with distilled water to remove unreacted GA. After that the activated beads were ready for immobilization step as shown in Scheme 1.

### 3.4. Enzyme Immobilization

The concentration of the enzyme reacted with the activated gel beads has an obvious effect. The reaction occurred between the free C=O group found on glutaraldehyde and the NH_2_ group found in the enzyme forming C=N– bond, Scheme 1.

### 3.5. Determination of Maximum Loading and Optimum Time of Immobilization Using 2^2^ Full-Factorial Central Composite Experiment Designs

In order to optimize the amount of loading units and the time of loading using gel beads treated with PEI and GA, 2^2^ full-factorial central composite design [18] was applied with four-star points (±∞) and three replicates at the center point. The coded and actual values are described in Table 1.

### 3.6. Optimum Lactose pH

The effect of pH on the activity of the free and immobilized *β*-gal enzyme was examined by incubation of 10 U of both enzyme forms in 10 mL of lactose solution 200 mM for 3 hrs at different pH values ranging from 2.2 to 8 pH at 37°C. The data was normalized to 100% relative activity at pH 5 for the free enzyme and at pH 6 for the immobilized enzyme. The relative activity at each pH is expressed as a percentage of the 100% activity.

### 3.7. Optimum Lactose Temperature

The optimum temperature for the free and immobilized *β*-gal was examined. 10 U of free and immobilized *β*-gal was incubated for 3 hrs into 10 mL lactose solution 200 mM at pH 6 and temperatures from 30 to 75°C. The optimum temperature has been taken as 100% activity and the relative activity at each temperature is expressed as a percentage of the 100% activity.

### 3.8. Kinetic Constants of Free and Immobilized *β*-Galactosidase

To determine the affinity of the enzyme for its substrate, *K*_*m*_ and *V*_max⁡_ can be determined by using free and immobilized *β*-gal. of 5 U for each and using different concentrations of lactose ranging from 25 to 200 mM at 37°C and pH 4.5 for 3 h.

### 3.9. Full Conversion

Full conversion of lactose, lactose in milk, and also lactose in whey was determined by using 10 U of immobilized and free enzyme incubated in 10 mL of each solution at nearly the same concentration of lactose at 37°C, pH 6 for 3 hrs.

### 3.10. Operational Stability

To study the reusability of immobilized *β*-galactosidase for lactose hydrolysis in milk, whey, and lactose solution, 10 mL of milk containing 8% lactose, 10 mL of whey, and 10 mL of 200 mM lactose were incubated at 37°C and pH 6 with 5 g of immobilized *β*-galactosidase containing 50 U.

## 4. Results and Discussion

### 4.1. Activation Process

Polyethyleneimine (PEI) is a synthetic cationic polymer that contains primary, secondary, and tertiary amine groups in its skeleton. PEI has been widely used to cross-link the gel surfaces before reaction with enzymes to avoid their leakages. Polyethyleneimine (PEI) is used as an activator and spacer to make the enzyme far away from beads to be free in its reaction with the substrate and thus it reacts with the negative charge on the beads surface by its positive charge to form ionic bond as shown in Scheme 1.

Glutaraldehyde is a cheap and very efficient cross-linker and hence remains the reagent of choice for cross-linking, although many reagents and newer methods are available [19]. Glutaraldehyde is a very reactive substance and the use of increasing concentrations of it to activate a support may result in matrices with different internal structure and also affect the surface of the matrices [20]. Glutaraldehyde reacts with aminated gel beads and replaces the free NH_2_ and turns it to N=C bond forming free C=O which the enzyme will attach to as shown in Scheme 1.

The results in Table 2 represent the design matrix of 11 trial experiments. For predicting the optimal point, a second-order polynomial function was fitted to correlate the relationship between independent variables (the amount of loading units and time of loading) and response of *β*-galactosidase activity, the polynomial equation was in the following form:

(1) Yβ-galactosidase=β0+β1X1+β2X2+β11X12+β22X22+βb12X1X2,

where *Y*_*β*-galactosidase_ is the predicted activity of *β*-galactosidase activity U/g beads, *X*_1_ and *X*_2_ are the independent variables corresponding to the loading time and amount of loading units, respectively, *β*_0_ is the intercept, *β*_1_ and *β*_2_, are linear coefficients, *β*_11_ and *β*_22_, are quadratic coefficients, and *β*_12_ is cross product coefficients. Statistical software SPSS (version 16.0) was used for the regression analysis of the experimental data obtained. The quality of fitting the polynomial model equation was expressed by the coefficient of determination *R* ^2^. Experiments were performed in triplicate and mean values were given.

The results obtained by 2^2^ full-factorial central composite design were analyzed by standard analysis of variance (ANOVA) as shown in Table 3. The second-order polynomial equation providing the levels of enzyme activity as a function of loading time (*X*_1_) and amount of loading units (*X*_2_) can be predicted by the following equation:

(2) Yβ-galactosidase=1.344−0.041X1+0.368X2+0.004X12−0.001X22−0.009X1X2.

As shown in Table 3, ANOVA for *β*-galactosidase activity (*Y*_Beads_) immobilized onto gel beads indicated the *F* value to be 14.549, which implied that the model is significant. Model terms having values of Prob > *F* (0.000) less than 0.05 were considered significant. ANOVA showed an *R* ^2^ of 0.980 which was equivalent to 98.0%. These results proved that the data variability could be explained by this model. The observed *R* ^2^ of 0.980 was in reasonable agreement with the adjusted *R* ^2^ of 0.960, which again ensured a satisfactory adjustment of the quadratic model to the experimental data. A value of 99.9% correlation between observed and predicted results was obtained as in Table 2 for the trial number 8 that showed the highest enzyme loaded capacity of 11.302 U/g gel beads compared to 11.443 U/g gel beads for the experimental and predicted values, respectively. All the above reflected the accuracy and applicability of the central composite design for optimization of *β*-galactosidase immobilization process. The maximum *β*-galactosidase loading capacity of 11.443 U/g beads was obtained by loading 40 U for 8 hours. This result was very similar to the predicted value obtained by the polynomial equation (11.357 U/g beads). The recovery of the enzyme activity was almost 29%. This result is somewhere reasonable compared to other authors who recovered between 2 and 83% using immobilized *β*-galactosidase from the same enzyme species [21].

**Figure 2:**
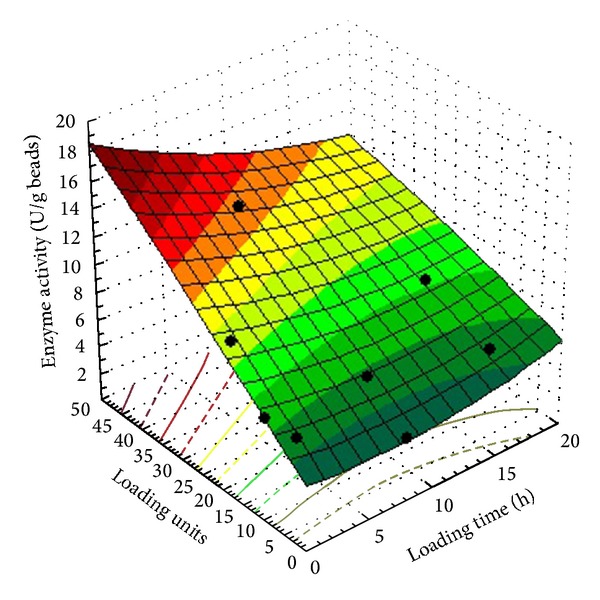
Effect of the loading time and amount of loading enzyme solution (units) on *β*-galactosidase activity (U/g beads).

Three-dimensional response surfaces were plotted on the basis of the model equation, to investigate the interaction among the variables and to determine the optimum value as shown in Figure 2. Figure 2 showed that higher levels of the *β*-galactosidase activity were attained with increasing the loading units and loading time till 8 hours which is sufficient for maximum enzyme loading.

### 4.2. Effect of Substrate pH

**Figure 3:**
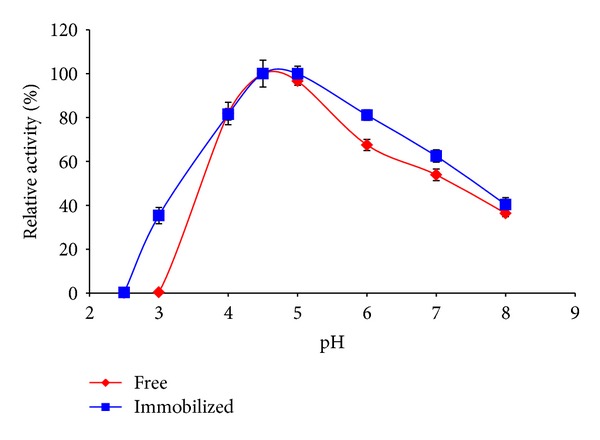
Optimum pH for free and covalently immobilized *β*-galactosidase onto gel beads treated with polyethylenimine (PEI), followed by glutaraldehyde using free lactose.

As shown from Figure 3 the optimum pH for free and immobilized *β*-gal enzyme was 4.5–5 pH, and it is also obvious that the immobilized enzyme is more stable than the free one and gives higher activity than the free one at each pH.

### 4.3. Effect of Substrate Temperature

**Figure 4:**
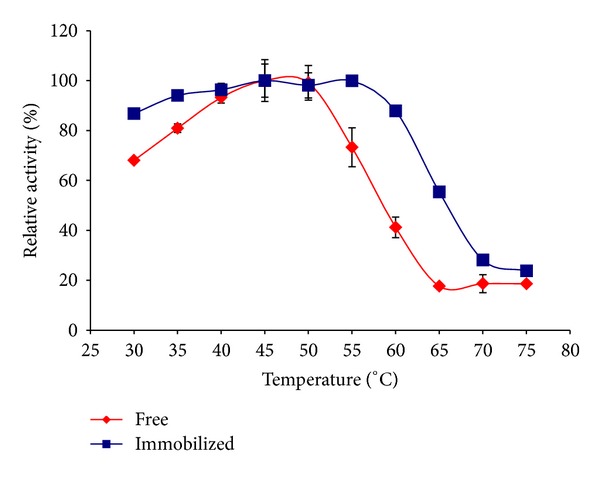
Optimum temperature for free and covalently immobilized *β*-galactosidase onto gel beads treated with polyethylenimine (PEI), followed by glutaraldehyde.

Figure 4 Illustrates the optimum temperature profile for the immobilized and free *β*-gal enzyme, and from the figure the immobilized enzyme was found to be stable at a wider range of temperature (45–55°C) than the free enzyme (45–50°C). The shift of the optimum temperature towards higher temperatures is an indication of the more thermal stability for the enzyme after immobilization process.

### 4.4. Kinetic Constants of Free and Immobilized *β*-Galactosidase

**Figure 5:**
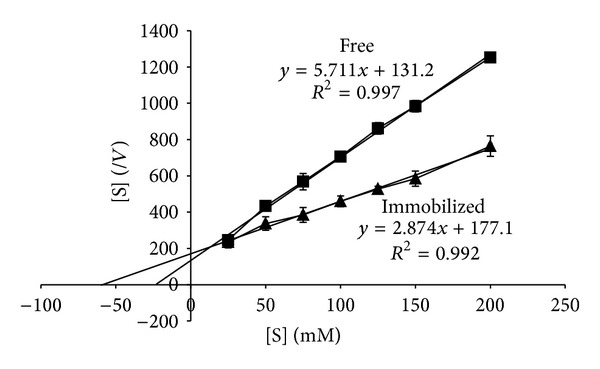
Kinetic parameters of free and immobilized *β*-galactosidase using the Hanes-Woolf plot method.

The kinetic constants of free and immobilized *β*-gal. were calculated using the Hanes-Woolf plot method as shown in Figure 5. The calculated values are shown in Table 4. The apparent *K*_*m*_ after immobilization, 61.6 mM, is higher than that of the free enzyme, 22.9 mM, which indicates that a higher concentration of substrate is needed for the immobilized enzyme compared to the free enzyme, while the maximum reaction velocity “*V*_max⁡_” value for the immobilized enzyme was higher than that of the free enzyme; that is, it increased from 131.2 *μ*mol·min^−1^ to 177.1 *μ*mol·min^−1^.

### 4.5. Full Conversion

**Figure 6:**
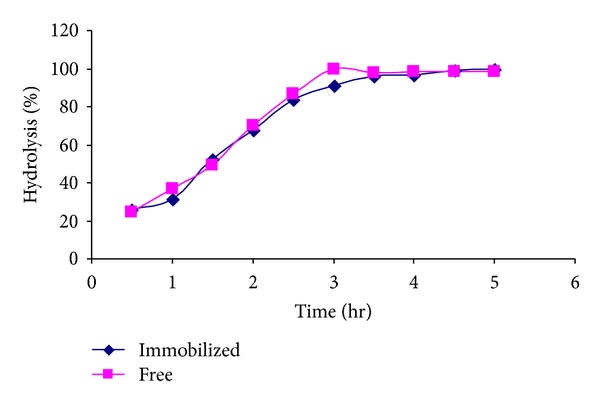
Lactose hydrolysis using free and immobilized *β*-galactosidase onto PEI treated gel beads.

As shown in Figure 6, the relative rate of conversion of lactose to glucose using the free enzyme was higher for the free enzyme than the immobilized one for the first 3.5 hours. After which, both enzyme forms reached their maximum relative conversion rate at around 4 h. The higher activity for the free enzyme compared to the immobilised form for the first 3.5 hours could be regarded to the time required for the immobilised enzyme to retain its better 3D conformation inside the gel beads.

**Figure 7:**
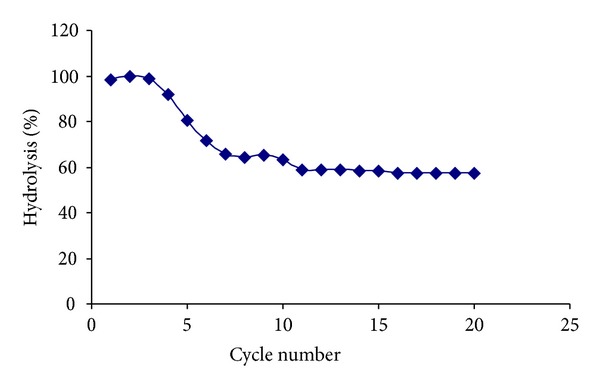
Reusability of immobilized *β*-galactosidase.

*Operational Stability*. The stability of immobilized enzyme, easiness of separation, and capability of being reused many times are more important and advantageous for industrial enzymes. As shown in Figure 7, the immobilized *β*-gal enzyme was used repeatedly 20 times and the residual activity was about 60% of its initial. This behavior may be related to the pH of the substrate medium.

## Figures and Tables

**Table 1:** Independent variables and the concentration levels studied in CCD.

Independent variables			Range of levels		
	−*∞*	−1	0	+1	+*∞*
Time (*X*_1_)	15 min	1	8	16	24
Units (*X*_2_)	1	5	10	20	40

**Table 2:** Experimental results of CCD for *β*-galactosidase activity.

Trial number	*X*_1_	*X*_2_	Experimental	Predicted
			*β*-galactosidase activity (U/g beads)	*β*-galactosidase activity (U/g beads)
1	−1	−1	3.733	2.867
2	−1	+1	8.159	7.877
3	+1	−1	3.442	2.597
4	1	1	5.848	5.582
5	−*∞*	0	4.537	4.681
6	+*∞*	0	3.616	3.874
7	0	−*∞*	0.988	1.357
8	0	+*∞*	11.443	11.302
9	0	0	3.965	3.922
10	0	0	3.965	3.922
11	0	0	3.965	3.922

**Table 3:** ANOVA test for the experiment.

Term	Response *Y*_Beads_ of
	*β*-galactosidase activity (U/g beads)
*F* value	48.945
*P* > *F*	0.000
*R*	0.990
*R* ^2^	0.980
Adjusted *R* ^2^	0.960
Standard error of the estimate	0.557155

**Table 4:** Kinetic constants of free and immobilized *β*-galactosidase.

*β*-Galactosidase form	Kinetic constants	
	*K*_*m*_ (mM)	*V*_max⁡_ (*µ*mol·min^−1^)
Free	22.9	131.2
Immobilized	61.6	177.1
